# Frankfurter Konzept einer stationären Cochleaimplantat-Frührehabilitation: Untersuchung zur Durchführbarkeit als Anschlussheilbehandlung

**DOI:** 10.1007/s00106-024-01440-z

**Published:** 2024-02-15

**Authors:** Stefanie Bruschke, Roland Zeh, Uwe Baumann, Silke Helbig, Timo Stöver

**Affiliations:** 1 Klinik für HNO-Heilkunde, Goethe-Universität Frankfurt, Universitätsklinikum, Frankfurt a. M, Deutschland; 2 MEDIAN Kaiserberg-Klinik, Bad Nauheim, Deutschland

**Keywords:** Cochleaimplantation, Anschlussheilbehandlung, Hörrehabilitation, Rehabilitationsergebnis, Sprachverstehen, Cochlear implantation, Follow-up treatment, Auditory rehabilitation, Rehabilitation outcome, Speech discrimination

## Abstract

**Hintergrund:**

Mit der im Jahr 2020 aktualisierten AWMF-Leitlinie zur Versorgung mit einem Cochleaimplantat (CI) wurde erstmals der gesamte Prozess einer CI-Versorgung definiert. In der vorliegenden Studie wurden die Machbarkeit und die Ergebnisse einer sehr frühen Rehabilitationsmaßnahme (Reha) untersucht.

**Methodik:**

Es wurden 54 Patienten in die Interventionsgruppe (IG) eingeschlossen, bei der die Reha innerhalb von 14 (maximal 28) Tagen nach der Implantation eingeleitet wurde. In eine Kontrollgruppe (KG, *n* = 21) wurden Patienten mit deutlich längerer Wartezeit eingeschlossen. Neben dem Beginn und der Dauer der Reha wurde das mit CI erreichte Sprachverstehen zu verschiedenen Zeitpunkten innerhalb von 12 Monaten erfasst. Zusätzlich wurde mit Fragebögen der Aufwand der Anpassung des CI-Prozessors und die Zufriedenheit der Patienten mit dem Ergebnis sowie dem Zeitpunkt des Beginns der Reha ermittelt.

**Ergebnisse:**

Die Wartezeit zwischen Implantation und Beginn der Reha lag in der IG bei 14 Tagen und in der KG bei 106 Tagen (Mediane). Es konnten 92,6 % der Patienten der IG die Reha innerhalb von 14 Tagen antreten. Der Effekt der Reha lag in der IG bei 35 und in der KG bei 25 Prozentpunkten (Freiburger Einsilbertest). Nach 6 und 12 Monaten (M) CI-Nutzung zeigten beide Gruppen sowohl in der Testbedingung in Ruhe (IG/KG 6M: 70 %/70 %; 12M: 70 %/60 %, Freiburger Einsilbertest) als auch im Störgeräusch (IG/KG 6M: −1,1 dB SNR/–0,85 dB SNR; 12M: −0,65 dB SNR/0,3 dB SNR, Oldenburger Satztest) vergleichbare Ergebnisse. Die mittels des Fragebogens Speech, Spatial and Qualities of Hearing Scale (SSQ) erfassten Ergebnisse für die Einschätzung der Hörqualität zeigten nach 6 Monaten eine bessere Bewertung in der IG, die sich nach 12 Monaten an die Ergebnisse der KG anglich. Die IG war mit dem Zeitpunkt des Beginns der Reha deutlich zufriedener als die KG. Alle anderen aus Fragebögen ermittelten Daten zeigten keine Unterschiede zwischen den beiden Gruppen.

**Schlussfolgerung:**

Der sehr frühe Beginn einer stationären Reha nach Cochleaimplantation ist erfolgreich umsetzbar. Die Reha konnte innerhalb von 7 Wochen nach der Implantation abgeschlossen werden. Der Vergleich der Ergebnisse der Tests des Sprachverstehens vor und nach der Reha zeigte eine deutliche Steigerung. Somit ist ein deutlicher Reha-Effekt nachweisbar. Die Aufnahme der CI-Rehabilitation in den Katalog der Anschlussheilbehandlungen ist somit wissenschaftlich begründet und damit dringend zu empfehlen.

## Abstract (en)

**Background:**

The Association of the Scientific Medical Societies in Germany (AWMF) clinical practice guideline on cochlear implant (CI) treatment, which was updated in 2020, defined the entire process of CI care for the first time. In the present study, the feasibility and results of very early rehabilitation were examined.

**Materials and methods:**

The intervention group (IG) comprised 54 patients in whom rehabilitation was initiated within 14 (maximally 28) days after implantation. Patients with a significantly longer waiting time were included in the control group (CG, *n* = 21). In addition to the start and duration of rehabilitation, the speech intelligibility achieved with CI was recorded at different timepoints within a 12-month period. In addition, questionnaires were used to assess the effort of fitting the CI processor and the patients’ satisfaction with the outcome as well as the timing of the start of rehabilitation.

**Results:**

Median waiting time between implantation and start of rehabilitation was 14 days in the IG and 106 days in the CG; 92.6% of IG patients were able to start rehabilitation within 14 days. The effect of rehabilitation in the IG was 35 and in the CG 25 percentage points (Freiburg monosyllabic test). After 6 and 12 months of CI use, both groups showed comparable results in the test condition in quiet (IG/CG 6 months: 70%/70%; 12 months: 70%/60%, Freiburg monosyllabic test) and in noise (IG/CG 6 months: −1.1–0.85 dB SNR; 12 months: −0.65 dB SNR/0.3 dB SNR, Oldenburg sentence test). Hearing quality assessment scores collected by SSQ (Speech, Spatial and Qualities of Hearing Scale) questionnaire showed better scores in the IG at 6 months, which converged to CG scores at 12 months. The IG was significantly more satisfied with the timing of the start of rehab than the CG. All other data obtained from questionnaires showed no differences between the two groups.

**Conclusion:**

A very early start of inpatient rehabilitation after cochlear implantation was successfully implemented. The rehabilitation was completed within 7 weeks of CI surgery. Comparison of speech recognition test results before and after rehabilitation showed a significant improvement. A clear rehabilitation effect can therefore be demonstrated. Inclusion of CI rehabilitation in the German catalog of follow-up treatments is thus scientifically justified and therefore strongly recommended.

Die Rehabilitation ist gemäß der im Jahr 2020 aktualisierten Leitlinie CI-Versorgung essenzieller Bestandteil der Cochleaimplantat(CI)-Versorgung. Gegenwärtig verzögert der i. d. R. zeitaufwendige Genehmigungsprozess für eine stationäre Rehabilitation die zeitnahe Wiedereingliederung der von einer schweren Hörstörung betroffenen Patienten. In der vorliegenden Arbeit wurde die Machbarkeit einer als Anschlussheilbehandlung (AHB) konzipierten sehr frühen stationären Rehabilitationsmaßnahme nach CI-Op. untersucht. Die vorgestellten Daten zeigen, dass 92,6 % der CI-Patienten die stationäre Hörrehabilitation innerhalb von 14 Tagen nach der Entlassung antreten konnten. Die Frührehabilitation nach CI-Versorgung („Frankfurter Konzept“) wurde somit erfolgreich als AHB evaluiert.

## Basis- und Folgetherapie nach CI-Versorgung

Das Cochleaimplantat (CI) ist eine Neuroprothese, die bei Patienten mit hochgradigem bis an Taubheit grenzendem Hörverlust eingesetzt wird [15, 16]. Durch die Nutzung eines CI kann die Verständlichkeit von Sprache signifikant verbessert [20] und die Lebensqualität in Alltagssituationen gesteigert werden [18].

Nach Indikationsstellung erfolgt die Implantation mit anschließender Basistherapie. Die Basistherapie umfasst die Erstanpassungsphase des CI-Audioprozessors mit Erstaktivierung des Systems und medizinischen sowie audiologischen Kontrollen. Anschließend erfolgt die Folgetherapie mit dem Ziel, eine best- und schnellstmögliche Nutzung des Implantatsystems zu erreichen. Die Folgetherapie setzt sich aus den Teilbereichen der audiologischen, hörtherapeutischen, sprachtherapeutischen und medizinischen Therapie zusammen [9]. Nach Abschluss der Folgetherapie schließt eine lebenslange Nachsorge an, um eine bestmögliche Kommunikationsfähigkeit dauerhaft sicherstellen zu können.

## Inhalte und Zeitpunkte einer CI-Rehabilitationsmaßnahme

Innerhalb dieses Versorgungsprozesses ist gemäß der im Jahr 2020 aktualisierten Leitlinie zur CI-Versorgung eine Rehabilitationsmaßnahme empfohlen [9]. Diese kann sowohl Teil der Basis- als auch der Folgetherapie sein. Die Rehabilitation umfasst therapeutische Inhalte, wie z. B. Hör- und Sprachtraining, Anpassungen des Prozessors, sowie Beratungsgespräche. In der Klinik der Autoren kann die Rehabilitation zeitlich zusammenhängend stationär in einer Rehaklinik erfolgen, i. d. R. über 3–5 Wochen, oder ambulant in einem entsprechenden Rehazentrum. Hier werden die ambulanten Termine über einen Zeitraum von mehreren Monaten verteilt. Die Patienten können frei wählen, ob sie die Rehabilitation ambulant oder stationär absolvieren möchten. In dieser Arbeit wurde ausschließlich stationäre Rehabilitation betrachtet, die in der MEDIAN Kaiserbergklinik Bad Nauheim durchgeführt wurde.

Zeh und Baumann (2015) wiesen bereits früher die Effektivität einer stationären Rehabilitationsmaßnahme auf die Verständlichkeit von Sprache mit CI nach. Nach Abschluss der Rehabilitation zeigte sich in verschiedenen Tests zur Prüfung des Sprachverstehens eine mittlere Verbesserung von etwa 20 Prozentpunkten [22].

Bislang gibt es in Deutschland kein einheitliches Konzept, das den Rehabilitationsprozess nach einer CI-Versorgung beschreibt. Die strukturelle Umsetzung der Basis- und Folgetherapie beruht meist auf individuellen, lokalen Konzepten der jeweiligen CI-versorgenden Einrichtung. So können Rehabilitationen stationär, ambulant oder gemischt durchgeführt werden. Für jeden einzelnen Fall ist bislang eine individuelle Beurteilung und Genehmigung durch den Kostenträger erforderlich. Aktuell muss hierzu ein Antrag auf Rehabilitation (Formular G100) beim zuständigen Kostenträger (Krankenversicherung, Rentenversicherung) gestellt werden. Die Rate der Ablehnungen ist bei Erwachsenen je nach Bundesland unterschiedlich. In Einzelfällen mussten CI-Patienten den Anspruch auf eine stationäre Rehabilitationsmaßnahme durch ein Sozialgerichtsverfahren erstreiten [4]. Dies führt zu einer nicht unerheblichen Verzögerung des Beginns der Rehabilitation.

Im Gegensatz zur CI-Rehabilitation wird in Deutschland unmittelbar anschließend an viele andere Operationen oder Erkrankungen, wie z. B. nach der Implantation von Endoprothesen oder Bypass-Operationen, eine stationäre Rehabilitation eingeleitet. Diese Rehabilitationsmaßnahmen werden als „Anschlussheilbehandlung“ (AHB) bezeichnet. Dabei ist ein Rehabilitationsbeginn innerhalb von 14 Tagen nach der Krankenhausentlassung eine unabdingbare Voraussetzung zur Einleitung einer AHB [8]. Für die CI-Versorgung ergibt sich damit die Fragestellung, ob auch nach der Implantation eine Rehabilitation bereits sehr früh, im Idealfall innerhalb von 14 Tagen nach der Krankenhausentlassung, möglich ist. Hierdurch würden die formalen Voraussetzungen einer „CI-AHB“ erfüllt werden.

Bislang wurde die Anpassung des CI-Audioprozessors i. d. R. erst nach einer Einheilungszeit von etwa 3–6 Wochen durchgeführt, sodass ein Rehabilitationsbeginn innerhalb der ersten 2 Wochen nach der Krankenhausentlassung gar nicht möglich war [12, 15]. Durch Verbesserungen der Op.-Technik, wie beispielsweise der Small-Incision-Technik [17], ist es aber inzwischen möglich, den CI-Audioprozessor bereits 2–3 Tage nach der Implantation zu aktivieren [1]. In vorausgegangenen Arbeiten konnte bereits gezeigt werden, dass die frühe Anpassung des CI-Audioprozessors innerhalb weniger Tage nach der Implantation erfolgreich umsetzbar ist und im Vergleich zur Anpassung nach der Standardeinheilungszeit zu äquivalenten Hörleistungen führt [5, 6, 11]. Die Anpassung des CI-Audioprozessors unmittelbar nach der Op. ermöglicht es damit auch, die Rehabilitation sehr früh nach der CI-Implantation zu beginnen. Ein entscheidender Vorteil der frühen Rehabilitation ist eine dadurch deutlich schneller mögliche Wiedereingliederung des Patienten in Beruf und Alltag. Bislang ist es in Deutschland aber nicht möglich, ohne Genehmigung des zuständigen Kostenträgers eine CI-Rehabilitation zu beginnen.

## Ziel der Studie

Das Ziel der hier beschriebenen Studie ist daher die Untersuchung der Machbarkeit des frühen Beginns einer stationären Rehabilitation innerhalb von 14 (maximal 28) Tagen nach der Implantation des CI. Die Ergebnisse wurden mit den Daten aus einer KG verglichen, bei der eine Rehabilitation nach dem bisherigen Standardprozess durchgeführt wird. Ermöglicht wurde dieses Pilotprojekt durch eine Kooperation zwischen der HNO-Universitätsklinik Frankfurt, der MEDIAN Kaiserberg-Klinik Bad Nauheim und verschiedenen Kostenträgern (Deutsche Rentenversicherung [DRV] Bund, DRV Hessen, Knappschaft Bahn/See, Deutsche Angestellten-Krankenkasse [DAK]). Hierdurch konnte der Beantragungsprozess für eine CI-Rehabilitation wesentlich vereinfacht und die Wartezeit für die Aufnahme in die Rehabilitationseinrichtung deutlich verkürzt werden.

Die Hauptfragestellung der Studie war, ob Patienten mit sehr kurzer CI-Hörerfahrung in gleicher Weise von einer Rehabilitationsmaßnahme profitieren können wie Patienten nach mehrmonatiger CI-Nutzung und damit längerer Gewöhnung.

## Material und Methode

### Patienten

Zur Untersuchung der Fragestellung wurden 2 Gruppen gebildet (Interventionsgruppe und Kontrollgruppe). In die Interventionsgruppe (IG) wurden 54 Patienten (23 m., 31 w.) eingeschlossen. In dieser Gruppe wurde die (stationäre) Rehabilitationsmaßnahme sehr früh (Ziel innerhalb von 14 Tagen, maximal 28 Tage) nach der CI-Op. begonnen. In die Kontrollgruppe (KG) wurden 21 Patienten (6 m., 15 w.) eingeschlossen, bei denen die Rehabilitationsmaßnahme postoperativ nach dem bisher üblichen Prozess über einen Antrag auf Rehabilitation bei den zuständigen Kostenträgern beantragt wurde. Als weitere Einschlusskriterien wurden ein Mindestalter von 18 Jahren sowie eine ein- oder beidseitige CI-Versorgung festgelegt. In das Patientenkollektiv konnten alle potenziellen CI-Kandidaten eingeschlossen werden, dazu zählten auch Patienten mit einseitiger Ertaubung (SSD, „single-sided deafness“). Die demografischen Daten der Patientengruppen sind in Tab. 1 dargestellt. Bei allen Studienteilnehmern wurde eine frühe Anpassung des CI-Audioprozessors innerhalb von 3 Tagen nach der CI-Op. durchgeführt [5, 11]. Unabhängig von der Zuordnung erfolgte in beiden Gruppen die bereits zuvor beschriebene stationäre Rehabilitationsmaßnahme zur Hörrehabilitation mit CI [22].

**Table 1:** 

	Interventionsgruppe	Kontrollgruppe
Alter (Mittelwert)	51,7 Jahre	52,9 Jahre
Minimum/Maximum	19/86 Jahre	43/65 Jahre
*Versorgungsart*		
Bilateral	15	9
Bilateral einzeitig	4	–
Bimodal	20	10
Unilateral	15	2
Dauer der Schwerhörigkeit (Mittelwert)	21,8 Jahre	27,3 Jahre
Minimum/Maximum	1/58 Jahre	5/50 Jahre
Hörgeräteerfahrung (Mittelwert)	20,9 Jahre	22,8 Jahre
Minimum/Maximum	1/50 Jahre	2/46 Jahre
*Implantate*		
HiFocus™ 3D Ultra SlimJ^a^	3	–
HiFocus™ 3D Ultra Mid-Scala^a^	1	–
CI512^b^	–	2
CI612^b^	17	10
CI622^b^	2	–
CI632^b^	6	2
Flex24^c^	–	1
Flex26^c^	10	3
Flex28^c^	14	3
FlexSoft^c^	1	–
*Prozessoren*		
CP1000^b^	18	6
CP1150^b^	5	3
CP950^b^	2	5
Naída CI M90^a^	2	–
Naída CI Q90^a^	2	–
Rondo 2^c^	1	–
Rondo 3^c^	11	1
Sonnet 2^c^	13	6

^a^Advanced Bionics, Valencia (CA), USA, ^b^Cochlear, Macquarie, Australien, ^c^MED-EL, Innsbruck, Österreich

### CI-Versorgungsprozess

Der CI-Versorgungsprozess an der Klinik der Autoren richtet sich nach den Empfehlungen der Leitlinie zur CI-Versorgung [3, 9] und beinhaltete im Rahmen der Studie folgende Teilbereiche:

- Indikationsstellung,
- Implantation,
- Basistherapie und
- Folgetherapie.

Der audiologisch-medizinische Teil der Basistherapie erstreckte sich über einen Zeitraum von maximal 2 Wochen nach der CI-Op. Er umfasste die Erstanpassungsphase des Prozessors sowie Wundkontrollen und wurde über 3 Termine verteilt in der Klinik durchgeführt. Der erste Termin fand 2–3 Tage nach der Implantation (Tag der Entlassung aus dem stationären Aufenthalt) statt und umfasste die Wundkontrolle sowie, nach Freigabe des Patienten, die Erstaktivierung des Prozessors. Bei 2 weiteren ambulanten Terminen wurden die Wundheilung weiter medizinisch kontrolliert, die Prozessoranpassung optimiert (audiologische Basistherapie) und die audiologische Diagnostik zur Erfassung des Hörerfolgs durchgeführt. Im Rahmen der Studie erfolgte die hör- und sprachtherapeutische Basis- und Folgetherapie in Form einer Anschlussheilbehandlung in der kooperierenden Reha-Einrichtung. Die Verlagerung der Hör‑/Sprachtherapie an eine externe Einrichtung entspricht den Vorgaben der derzeit geltenden CI-Leitlinie [3] und dem Weißbuch CI-Versorgung [9], da es sich um einen durch die CI-versorgende Einrichtung delegierbaren Anteil des Versorgungsprozesses handelt. Die Rehabilitation erfolgte extern als stationäre Rehabilitation in der Kaiserbergklinik Bad Nauheim. Die vom Kostenträger genehmigten Rehabilitationstage wurden zusammenhängend absolviert. In der Rehabilitation wurden therapeutische Maßnahmen durchgeführt, dazu zählten u. a. Hörtraining, Anpassungen des Audioprozessors, Beratungen, Einzeltherapien und Gruppentherapien. Im Rahmen der Folgetherapie erfolgten ambulante Termine an der CI-versorgenden Einrichtung (HNO-Klinik) 3, 6 und 12 Monate nach der CI-Versorgung. Hier wurde die medizinische und audiologische Folgetherapie durchgeführt. Um die frühe Rehabilitation mit dem Standardbeginn zu vergleichen, wurde der Rehabilitationszeitraum beider Gruppen erfasst. Hierzu wurde die Wartezeit zwischen Implantation und Rehabilitationsbeginn sowie die Dauer der am Stück durchgeführten stationären Rehabilitation dokumentiert.

Um die Therapieergebnisse beider Studiengruppen vergleichen zu können, wurde die Verständlichkeit von Sprache bei Nutzung des CI ermittelt. Dazu wurde das Sprachverstehen in Ruhe mittels des Freiburger Einsilbertests [13] bei 65 dB SPL im Freifeld ermittelt. Die Messung erfolgte monaural, bei nutzbarem Restgehör auf dem Gegenohr wurde ein Einsteckhörer zur Maskierung mit einem Breitbandrauschsignal verwendet (Pegel 70 dB HL). Darüber hinaus wurde die Sprachverständlichkeit im Störgeräusch entweder mithilfe des HSM(Hochmair-Schulz-Moser)-Satztests [14] bei einem Sprachpegel von 65 dB SPL und einem Signal-Rausch-Abstand von +10 dB geprüft (Testbedingung in der Reha-Einrichtung) oder der Oldenburger Satztest (OlSa) [21] bei einem festen Sprachpegel von 65 dB SPL und adaptivem Rauschpegel durchgeführt (Testbedingung in der HNO-Universitätsklinik). Beide Messungen im Störgeräusch erfolgten monaural im Freifeld mit Sprach- und Rauschsignal von vorn (S_0_N_0_-Bedingung). Bei nutzbarem Restgehör wurde das Gegenohr mit einem Ohrstöpsel und Kapselgehörschutz (Peltor, Fa. 3M, Neuss, Deutschland) doppelt geblockt. Um die subjektive Hörempfindung der Patienten zu evaluieren, wurden der Fragebogen Speech, Spatial and Qualities of Hearing Scale (SSQ) [10] und der Hearing Implant Sound Quality Index (HISQUI) Fragebogen [2] eingesetzt. Über einen selbst entwickelten Fragebogen wurde die subjektive Zufriedenheit der Patienten im Hinblick auf den Zeitpunkt des Rehabilitationsbeginns erfasst. Dazu wurde eine Likert-Skala mit 5 Stufen (sehr zufrieden, zufrieden, mäßig, eher unzufrieden, nicht zufrieden) verwendet. Mithilfe eines vom audiologischen Fachpersonal auszufüllenden Fragebogens wurde der Anpassaufwand mittels einer 3‑stufigen Likert-Skala (kurzer, mittlerer, langer Aufwand) eingeschätzt, sowie die mittlere Prozessornutzungsdauer über die Erfassung des Dataloggings der entsprechenden Anpass-Software dokumentiert.

**Figure 1:**
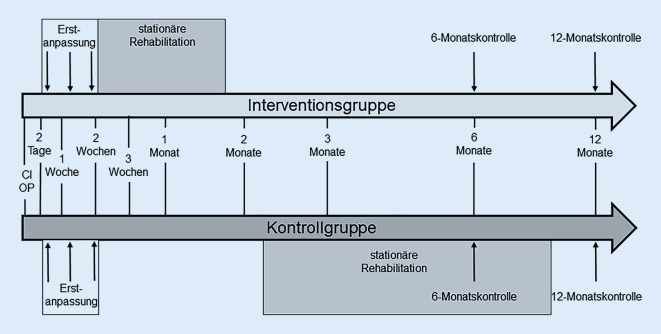

Die Datenerhebung erfolgte präoperativ (prä-Op.), zur Erstanpassung (EA) des CI-Audioprozessors, bei Aufnahme und Entlassung aus der Rehabilitation und zu den regulären klinischen Kontrollterminen nach 6 und 12 Monaten (6M/12M) nach CI-Op. Der Studienablauf ist in Abb. 1 schematisch dargestellt.

### Statistik

Die Daten wurden nach vorheriger Verifikation auf Normalverteilung (Kolmogorov-Smirnov-Test, *p* > 0,05), mithilfe parametrischer Tests, wie gepaarter T‑Test (einseitig) bzw. 2‑faktorielle Repeated-Measures(RM)-ANOVA mit Bonferroni-Korrektur für Mehrfachvergleiche, auf signifikante Unterschiede hin analysiert. Wenn keine Normalverteilung vorlag, wurde der Mann-Whitney-U-Test (MWU) bei Vergleichen zwischen den Gruppen verwendet, beim Vergleich innerhalb der Gruppen der Wilcoxon-Rangsummentest (WRS). Zur statistischen Auswertung wurde ein Signifikanzniveau von *p* *=* 0,05 festgelegt. Die Datenanalyse wurde mit SPSS Statistics 24.0 (Fa. IBM Corporation, Endicott/NY, USA) durchgeführt. Alle im Ergebnisteil genannten Zahlenwerte sind Zentralwerte (Mediane).

## Ergebnisse

### Rehabilitationszeitraum

**Figure 2:**
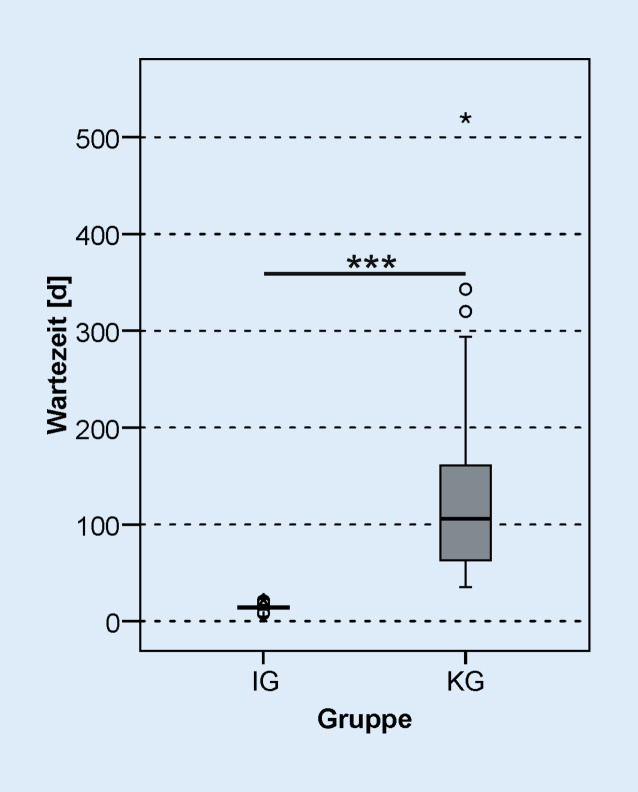

**Figure 3:**
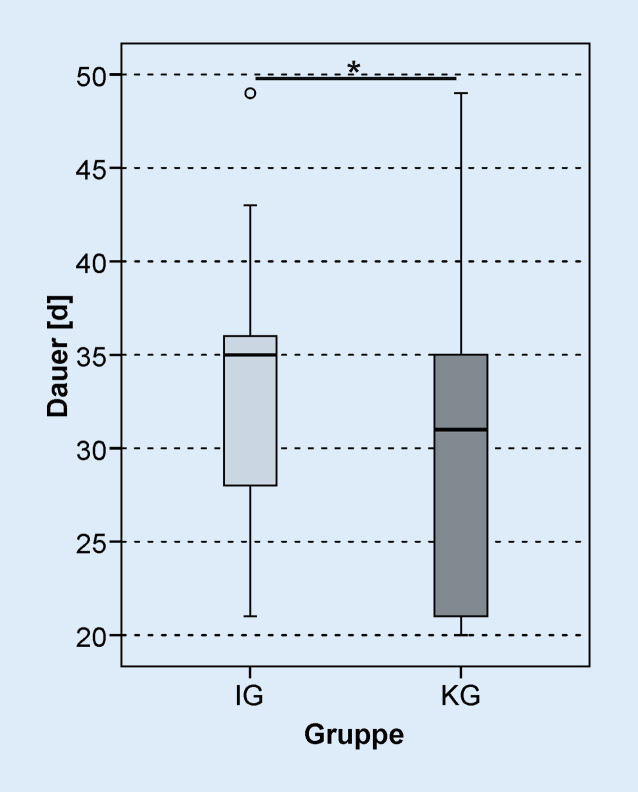

Die Wartezeit zwischen CI-Op. und dem Beginn der stationären Rehabilitation war in der IG signifikant kürzer als in der KG (*z*_*MWU*_ *=* −6,827;* p*_*MWU*_ < 0,001, Abb. 2). In der IG betrug die Wartezeit 14 Tage (Minimum 8 Tage, Maximum 23 Tage), die KG konnte nach 106 Tagen (Minimum 35 Tage, Maximum 520 Tage) die Rehabilitation beginnen. Bei 2 Patienten der IG betrug die Wartezeit auf die Rehabilitation 8 Tage. Beide Patienten haben alle 3 Erstanpassungstermine in kurzer Zeit absolviert und somit die audiologische Basistherapie in der Klinik abgeschlossen. Insgesamt konnten 92,6 % der Patienten der IG die Rehabilitation innerhalb von 14 Tagen antreten. Auch bei der Dauer der stationären Rehabilitation (Abb. 3) zeigte sich ein signifikanter Unterschied zwischen den Studiengruppen (IG: 35 Tage, KG: 31 Tage, *z*_*MWU*_ = −2,226; *p*_*MWU*_ = 0,026). Die IG konnte die Rehabilitation nach 49 (Minimum 29, Maximum 63) Tagen nach CI-Versorgung abschließen; die KG hat die Rehabilitation hingegen erst nach 129 (Minimum 69, Maximum 555) Tagen abgeschlossen.

### Sprachverstehen in Ruhe

**Figure 4:**
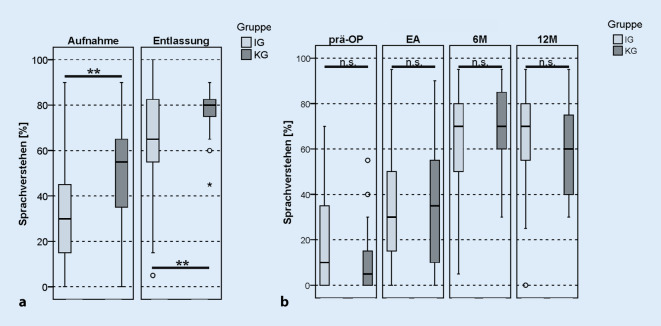

Beide Gruppen profitierten von der CI-Versorgung und wiesen postoperativ im Vergleich zum präoperativen Ergebnis ein signifikant verbessertes Einsilberverstehen auf (IG *z*_*WRS*_ = −3,799; *p*_*WRS*_ < 0,001; KG *T*_*T_paired*_ = −6,183; *p*_*T_paired*_ < 0,001). Der im Vergleich der Ergebnisse bei Aufnahme und Entlassung ermittelte Effekt der Rehabilitation zeigte, dass die IG mit 35 Prozentpunkten stärker profitierte als die KG, die eine Verbesserung von 25 Prozentpunkten aufwies. Dieser Unterschied in der Verbesserung des Sprachverstehens ist allerdings nicht statistisch signifikant (*T*_*T_unpaired*_ = 1,386; *p*_*T_unpaired*_ = 0,170). Das präoperativ und zu den 6M/12M-Intervallen erhobene Einsilberverstehen zeigte keinen signifikanten Unterschied zwischen beiden Gruppen (IG/KG 6M 70 %/70 %, *T*_*T_unpaired*_ = −0,716; *p*_*T_unpaired*_ = 0,477; 12M 70 %/60 %, *T*_*T_unpaired*_ = 0,731; *p*_*T_unpaired*_ = 0,469; Abb. 4b). Die KG zeigte gegenüber der IG sowohl bei Aufnahme als auch bei Entlassung aus der Rehabilitation ein signifikant besseres Einsilberverstehen (IG/KG Aufnahme 30 %/55 %, *T*_*T_unpaired*_ = −3,075; *p*_*T_unpaired*_ = 0,003; Entlassung 65 %/80 %, *T*_*T_unpaired*_ = −2,832; *p*_*T_unpaired*_ = 0,006; Abb. 4a).

### Sprachverstehen im Störgeräusch

**Figure 5:**
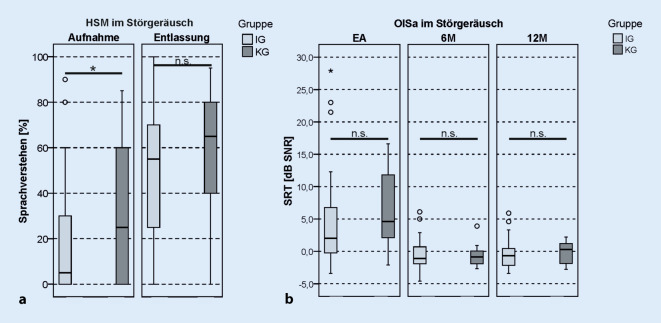

Beide Gruppen zeigten bei der Prüfung des Sprachverstehens im Störgeräusch im Vergleich der Ergebnisse zwischen Aufnahme und Entlassung aus der Rehabilitation eine signifikante Verbesserung (Aufnahme/Entlassung: IG 5 %/55 %, *z*_*WRS*_ = −5,506; *p*_*WRS*_ < 0,001; KG 25 %/65 %, *T*_*T_paired*_ = −4,558; *p*_*paired*_ < 0,001; Abb. 5a). Ebenso wie bei der Prüfung des Sprachverstehens in Ruhe erreichte die IG einen um 10 Prozentpunkte größeren Gewinn nach Abschluss der Rehabilitation. Dieser war allerdings nicht statistisch signifikant (*T*_*T_unpaired*_ = 0,971; *p*_*T_unpaired*_ = 0,335). Zum 6M-Intervall wies sowohl die IG als die KG eine deutliche Verbesserung der OlSa-SRT-Ergebnisse im Vergleich zur Messung bei Erstanpassung des CI-Audioprozessors auf (EA/6M: IG IG: 2,0 dB SNR/-1,1 dB SNR; KG 4,6 dB SNR/-0,85 dB SNR; Abb. 5b). Im 12M-Intervall lag das Ergebnis in beiden Gruppen unverändert zu den 6M-Ergebnissen (6M/12M: IG −1,1 dB SNR/–0,65 dB SNR; *z*_*WRS*_ = −0,278; *p*_*WRS*_ = 0,781; KG −0,85 dB SNR/0,3 dB SNR; *T*_*T_paired*_ = 2,187; *p*_*T_paired*_ = 0,094).

Es zeigten sich im Gruppenvergleich bei der Aufnahme in die Rehabilitation signifikant bessere Ergebnisse im HSM-Satztest im Störgeräusch in der KG (KG: 25 %, IG: 5 %, *z*_*MWU*_ = −2,035; *p*_MWU_ = 0,042), die sich bei der Entlassung anglichen (IG: 65 %, KG: 55 %, *T*_*T_unpaired*_ = −1,327; *p*_*T_unpaired*_ = 0,189).

### Subjektive Hörempfindung

**Figure 6:**
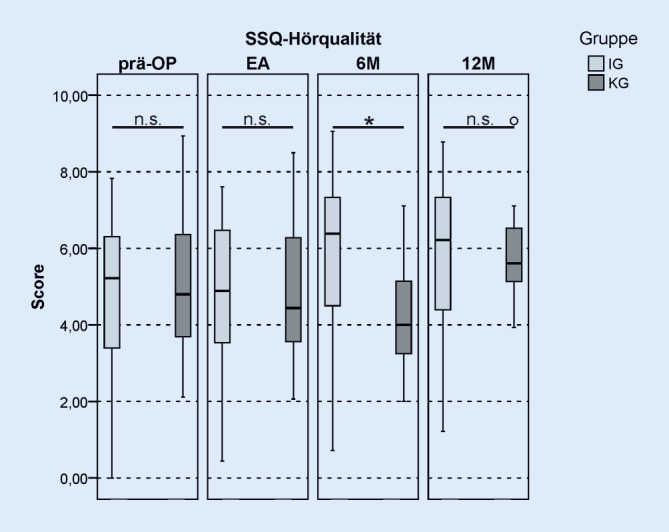

Der SSQ-Fragebogen erfasst die subjektive Hörempfindung in 3 Teilbereichen: Sprachverständlichkeit, räumliches Hören und Hörqualität. Die Ergebnisse des Fragebogens für die 3 Teilbereiche sind in Tab. 2 dargestellt. Im Bereich Hörqualität zeigte sich zum 6M-Intervall ein signifikanter Unterschied zwischen IG und KG, wobei die Hörqualität in der IG besser bewertet wurde als in der KG (Abb. 6). Nach 12 Monaten CI-Nutzung glich sich dieser Unterschied zwischen den Gruppen an, und es zeigte sich kein signifikanter Unterschied mehr. In den Bereichen Sprachverständlichkeit und räumliches Hören war zu keinem Zeitpunkt ein signifikanter Unterschied zwischen den Gruppen zu verzeichnen (Tab. 2). Es zeigte sich eine Tendenz, dass die Probanden der IG im 6M-Intervall etwas günstigere Bewertungen als die KG abgaben. Die Ergebnisse des HISQUI-Fragebogens wiesen keinen signifikanten Unterschied zwischen IG und KG zu allen Beobachtungszeitpunkten auf (Tab. 3). Es zeigte sich auch in den Ergebnissen des HISQUI-Fragebogens die Tendenz, dass die IG im 6M-Intervall eine höhere Bewertung der Hörqualität als die KG angab.

**Table 2:** 

**Bereich Hörqualität**				
Zeitpunkt	Score IG	Score KG	*T*_*T_unpaired*_	*p*_*T_unpaired*_
Präop.	5,2	4,8	−0,723	0,472
EA	4,9	4,4	0,236	0,814
6M	6,4	4,0	2,040	0,047
12M	6,2	5,6	−0,015	0,988
**Bereich Sprachverständlichkeit**				
Zeitpunkt	Score IG	Score KG	*T*_*T_unpaired*_	*p*_*T_unpaired*_
Präop.	3,7	3,4	−4,11	0,683
EA	4,6	3,6	0,851	0,399
6M	4,9	4,0	1,626	0,111
12M	5,4	4,6	0,479	0,635
**Bereich Hörqualität**				
Zeitpunkt	Score IG	Score KG	*T*_*T_unpaired*_	*p*_*T_unpaired*_
Präop.	2,4	3,5	−1,634	0,108
EA	3,9	3,8	−0,285	0,776
6M	5,1	3,9	0,816	0,419
12M	5,3	5,2	−0,125	0,901

*Präop.* präoperativ, *EA* nach abgeschlossener Erstanpassung, *6M* zur 6‑Monats-Kontrolle, *12M* 12-Monats-Kontrolle, *IG* Interventionsgruppe, *KG* Kontrollgruppe

Angabe statistischer Werte: T‑Wert (*T*_*T_unpaired*_) und *p*-Wert (*p*_*T_unpaired*_) nach t‑Test für ungepaarte Stichproben

**Table 3:** 

Zeitpunkt	Punkte IG	Punkte KG	*T*_*T_unpaired*_	*p*_*T_unpaired*_
Präop.	69,0	59,5	−0,128	0,889
EA	71,0	71,0	1,006	0,319
6M	81,0	65,0	1,128	0,265
12M	81,0	82,0	0,121	0,904

*Präop.* präoperativ, *EA* nach abgeschlossener Erstanpassung, *6M* 6-Monats-Kontrolle, *12M* 12-Monats-Kontrolle, *IG* Interventionsgruppe, *KG* Kontrollgruppe

Angabe statistischer Werte: T‑Wert (*T*_*T_unpaired*_) und *p*-Wert (*p*_*T_unpaired*_) nach t‑Test für ungepaarte Stichproben

### Audiologischer Fragebogen

Die Untersuchung des zeitlichen Aufwands der Anpassung des CI-Audioprozessors wies keinen signifikanten Unterschied zwischen den Studiengruppen auf (EA: *z*_*MWU*_ = −0,517; *p*_*MWU*_ = 0,605; 6M: *z*_*MWU*_ = −0,563; *p*_*MWU*_ = 0,573; 12M: *z*_*MWU*_ = −0,213; *p*_*MWU*_ = 0,831). Bei allen Terminen benötigte die Mehrzahl der Patienten (IG: 58,6–78,8 %, KG: 50–92,3 %) einen mittleren Anpassaufwand. Die Ergebnisse zeigten außerdem eine vergleichbare mittlere Prozessornutzungsdauer zwischen der IG (EA: 11,9 h/Tag, 6M: 13,5 h/Tag, 12M: 13,0 h/Tag) und KG (EA: 10,3 h/Tag, 6M: 13,8 h/Tag, 12M: 13,7 h/Tag) zu allen Untersuchungszeitpunkten (EA: *z*_*T_unpaired*_ = 1,388; *p*_*T_unpaired*_ = 0,178; 6M: *z*_*MWU*_ = −0,641; *p*_*MWU*_ = 0,522; 12M: *T*_*T_unpaired*_ = −0,167; *p*_*T_unpaired*_ = 0,868).

### Subjektive Zufriedenheit mit dem Zeitpunkt der Rehabilitation

Die Patienten der IG zeigten zum 12M-Intervall ein sehr hohes Maß an subjektiver Zufriedenheit mit dem Zeitpunkt der früh eingeleiteten Rehabilitation. Dabei waren 88,5 % „sehr zufrieden“ und 11,5 % „zufrieden“ mit dem Zeitpunkt des Rehabilitationsbeginns. Die Bewertungen des Beginns der Rehabilitation innerhalb der KG war signifikant schlechter als in der IG (*z*_*MWU*_ = −2,583; *p*_*MWU*_ = 0,01). In dieser Gruppe waren nur 50 % „sehr zufrieden“, 30 % zufrieden, 10 % mäßig zufrieden und 10 % unzufrieden.

## Diskussion

Anhand der Studienergebnisse wurde gezeigt, dass eine sehr früh eingeleitete Rehabilitation nach CI-Op. erfolgreich durchgeführt werden kann. Aus den Ergebnissen konnten keine Nachteile gegenüber dem bisher üblichen Prozess der Rehabilitation erhoben werden. Patienten mit sehr kurzer CI-Hörerfahrung profitieren in gleicher Weise von der Rehabilitationsmaßnahme wie Patienten nach mehrmonatiger CI-Nutzung.

### Rehabilitationszeitpunkt

Durch die bereits 2 Tage nach der Implantation begonnene Basistherapie, in Verbindung mit der Beschleunigung des Antrags- und Genehmigungsprozesses, konnte die Wartezeit auf den Beginn der stationären Rehabilitationsmaßnahme auf etwa 2 Wochen verkürzt werden. In der IG konnten 92,6 % der Patienten die Rehabilitation innerhalb von 14 Tagen antreten. Die restlichen 7,4 % der Patienten haben die Rehabilitation innerhalb von 23 Tagen begonnen. Gründe für den späteren Rehabilitationsbeginn war u. a. das Auftreten einer postoperativen Schwellung, die einen Druckverband für mehrere Tage erforderte. Dies führte zu einer Verschiebung der Erstanpassung um einige Tage und somit auch zu einer Verzögerung des Rehabilitationsbeginns. In anderen Fällen wurde die Rehabilitation aus persönlichen oder organisatorischen Gründen (z. B. Feiertage) nicht innerhalb von 14 Tagen nach der CI-Versorgung begonnen. In der IG konnte die Rehabilitation im Durchschnitt bereits 7 Wochen nach der CI-Versorgung abgeschlossen werden, während die meisten Patienten der KG zu diesem Zeitpunkt immer noch auf den Beginn der Rehabilitation warteten. Trotz des sehr frühen Beginns der Rehabilitation benötigte die IG für den Abschluss der Maßnahme im Schnitt nur minimal länger (4 Tage).

### Sprachtestergebnisse

Beide Studiengruppen zeigten nach Abschluss der Rehabilitation eine signifikante Verbesserung des Sprachverstehens sowohl in Ruhe als auch im Störgeräusch. Dabei war die Verbesserung des Sprachverstehens vom Beginn bis zum Abschluss der Rehabilitation in der IG um 10 Prozentpunkte (Mittelwert) größer als in der KG. Dies könnte darauf zurückzuführen sein, dass die KG bei der Aufnahme signifikant bessere Testergebnisse zeigte als die IG und deshalb ein etwas geringerer Zugewinn durch die Rehabilitation erreicht wurde.

Bei der Interpretation der Aufnahme- und Entlassungsergebnisse muss berücksichtigt werden, dass die Messzeitpunkte in der IG und KG stark voneinander abwichen. Die IG erzielte 7 Wochen nach Implantation und Abschluss der Rehabilitation sowohl in Ruhe als auch bei Störgeräuschen bessere Ergebnisse als die KG nach 15 Wochen (zu Beginn der Rehabilitation). Dies ist für alltagsrelevante Situationen bedeutsam. Die Ergebnisse belegen damit, dass das Frankfurter Konzept CI-Patienten deutlich früher eine optimale Nutzung des CI ermöglicht und so früher eine Verbesserung des Sprachverstehens erreicht.

Sowohl bei Aufnahme als auch bei Entlassung aus der Rehabilitationsmaßnahme zeigte die KG ein besseres Einsilberverstehen als die IG. Bei der Interpretation der Ergebnisse ist zu beachten, dass die KG zu diesem Zeitpunkt bereits eine deutlich längere Hörerfahrung mit dem CI hatte als die IG. Während Patienten der IG den CI-Audioprozessor vor Beginn der Rehabilitation nur wenige Tage nutzen konnten, konnte die KG bereits einige Wochen oder sogar Monate vor der Rehabilitation Hörerfahrungen sammeln. Beide Studiengruppen zeigten eine ausreichende und vergleichbare durchschnittliche Prozessornutzungsdauer über die Zeit [7]. Eine konsequente Nutzung des CI im Alltag scheint damit auch ohne eine strukturierte Rehabilitationsmaßnahme zu einer Verbesserung des Sprachverstehens zu führen. Durch die Rehabilitation verbessert sich die Verständlichkeit von Sprache aber zusätzlich signifikant. Die vorliegenden Ergebnisse stehen damit im Einklang zu früheren Untersuchungen, die ebenfalls den positiven Effekt der Rehabilitation auf die CI-Nutzung nachweisen konnten [22].

Die Ergebnisse zum 6M- und 12M-Intervall zeigten sowohl für Sprachtestergebnisse in Ruhe als auch im Störgeräusch eine vergleichbare Entwicklung in beiden Gruppen, nachdem die stationäre Rehabilitationsmaßnahme abgeschlossen war. Zu keinem Zeitpunkt konnten signifikante Unterschiede zwischen den beiden Gruppen nachgewiesen werden. Somit ist anhand der von den Autoren erhobenen Daten auch bei sehr frühem Beginn ein vergleichbarer Effekt der Rehabilitationsmaßnahme nachweisbar.

Bei 92,6 % der Patienten konnte die Rehabilitation innerhalb von 14 Tagen nach der Operation beginnen. Der Vergleich der IG mit der KG zeigte keine signifikanten Unterschiede im Sprachverstehen in Ruhe und im Störgeräusch, weder bei Aufnahme und Entlassung aus der Rehabilitation noch nach 6 Monaten CI-Nutzung. Demnach können bei einem Rehabilitationsbeginn innerhalb von 14 Tagen nach der CI-Versorgung vergleichbare Sprachtestergebnisse erzielt werden.

### Ergebnisse des SSQ-Fragebogens

Die Ergebnisse des SSQ-Fragebogens zum 6M-Intervall zeigten, dass die IG im Bereich der Hörqualität signifikant bessere Bewertungen als die KG abgab. Die IG scheint demnach subjektiv früher von der Rehabilitation zu profitieren als die KG. Nach 6 Monaten hatten 40 % der Patienten der KG die Rehabilitation noch nicht abgeschlossen. Die Ergebnisse des SSQ-Fragebogens in den Bereichen Sprachverstehen und räumliches Hören sowie die Daten des HISQUI-Fragebogens zeigten zu keinem Untersuchungszeitpunkt einen signifikanten Unterschied zwischen den Gruppen. Allerdings war hier eine Tendenz zu erkennen, dass die IG zum 6M-Intervall bessere Bewertungen als die KG vornahm.

### Zufriedenheit mit dem Zeitpunkt der Rehabilitation

Die sehr früh eingeleitete Rehabilitation wurde seitens der Patienten sehr positiv bewertet. Alle Patienten der IG zeigten ein hohes Maß an Zufriedenheit mit dem Zeitpunkt des Rehabilitationsbeginns. In der KG gab es hingegen einige Patienten, die mit dem Rehabilitationsbeginn nur mäßig bis nicht zufrieden waren. Dies ist vermutlich durch die teilweise sehr langen Wartezeiten zwischen Implantation und Rehabilitation von bis zu annähernd einem Jahr bedingt.

### Nutzung eines Single-Unit-Prozessors

Bei Erstaktivierung des Prozessors wurde bei insgesamt 27 Patienten (33 % der IG und 43 % der KG) ein Single-Unit-Prozessor verwendet, der direkt über dem Implantat getragen wird. In 22 Fällen gab es keine Probleme bei der Erstaktivierung, meist wurde ein starker Magnet verwendet, der im weiteren Verlauf der technischen Kontrollen abgeschwächt werden konnte. In 2 Fällen (einmal IG und einmal KG) war der Magnethalt sehr schwach, aber mit Nutzung eines Stirnbands war das Tragen des Single-Unit-Prozessors möglich. In 2 weiteren Fällen (2-mal IG) war der Magnethalt des Single-Unit-Prozessors bei Erstaktivierung zu schwach, sodass temporär auf ein Hinter-dem-Ohr-Gerät (HdO) gewechselt wurde. Am Ende der Basistherapie konnte dann wieder auf den Single-Unit-Prozessor zurückgewechselt werden. In einem Fall (KG) wurde kein ausreichender Halt mit dem Single-Unit-Prozessor erreicht, sodass dauerhaft auf ein HdO-Gerät gewechselt werden musste. Es war kein Unterschied hinsichtlich des Halts von Single-Unit-Prozessoren zwischen den Gruppen zu beobachten. Die frühe Basistherapie und Frührehabilitation scheint somit nicht in einem höheren Umfang die Nutzung von HdO-Prozessoren zu erfordern.

### Organisationskonzept der Rehabilitation

Das in dieser Studie verfolgte Konzept der stationären Rehabilitation von CI-Patienten wurde bereits in einer vorangegangenen Publikation in Form und Inhalt vorgestellt [22]. Neben diesem Organisationskonzept der Rehabilitation existieren in Deutschland weitere alternative Konzepte. Insbesondere wird an vielen Orten eine ambulante Rehabilitation von CI-Trägern verfolgt. Vermutlich lässt sich das Modell des sehr frühen Beginns der Rehabilitation auch auf andere Konzepte übertragen. Ein Vergleich der Konzepte war aber nicht das Ziel der vorliegenden Studie.

### CI-Rehabilitation als AHB

In der vorliegenden Studie konnte nachgewiesen werden, dass mehr als 90 % der Patienten der Interventionsgruppe spätestens 14 Tage nach Entlassung eine Rehabilitationsmaßnahme angetreten haben. Hierdurch konnte belegt werden, dass die weit überwiegende Anzahl der Patienten die formalen Kriterien zur Einordnung der postoperativen Rehabilitation (Folgetherapie) als AHB erfüllen. Die Respektierung der engen Zeitvorgabe einer AHB setzt die Erfüllung wichtiger Randbedingungen voraus. Zum einen muss vor Beginn der stationären Rehabilitation die Basistherapie in der implantierenden Einrichtung erfolgt sein. Die aktualisierte CI-Leitlinie fordert eine medizinische Kontrolle und audiologische Basistherapie im Rahmen der Inbetriebnahme des CI-Systems in der CI-versorgenden Einrichtung (CIVE) [3]. Dieser Prozessablauf wird demnach auch in den Vorgaben der CIVE-Zertifizierung dargestellt [19].

Aus diesem Grund ist die Basistherapie als tragender Bestandteil des CI-Versorgungsprozesses nicht delegierbar und muss in der implantierenden Einrichtung durchgeführt werden. Die Inbetriebnahme einer aktiven implantierten Neuroprothese liegt in der ärztlichen Verantwortung des Betreibers des Medizinprodukts (Medizinproduktbetreiberverordnung). Hierzu muss zunächst ärztlich festgestellt werden, dass der betroffene Patient überhaupt die medizinischen, audiologischen und psychologischen Voraussetzungen erfüllt, um eine postoperative Basistherapie beginnen zu können. Eine Reihe von Faktoren oder unerwarteten Ereignissen können den Beginn der Basistherapie nicht nur infrage stellen, sondern den Patienten sogar gesundheitlich gefährden. Die Voraussetzungen müssen daher kontinuierlich vor und auch während der Basistherapie beurteilt werden, da sie sich gerade unmittelbar postoperativ stark verändern können. Beispielhaft sind hier die ärztliche Beurteilung der Wunde u. a. in Bezug auf Wundinfektionen, Schwellung der Wunde, Schwellung der Spulenregion und der Hautdicke, die Beurteilung der Andruckkraft des Sendespulenmagnets, mögliche Druckstellen oder Hautnekrosen sowie Schmerzempfindungen. Zusätzlich muss die psychische Situation des Patienten nach der Op. beurteilt werden. Erst nach einer individuellen medizinischen Beurteilung der Eignung kann die ärztliche Freigabe eines Patienten zur audiologischen Basistherapie erfolgen. Im Rahmen der audiologischen Basistherapie muss die grundsätzliche Funktion des Implantatsystems sowie eine den Erfordernissen gerecht werdende Anpassung des CI-Prozessors sichergestellt werden.

Der Beginn der Basistherapie ist damit keinesfalls als Automatismus zu betrachten, sondern bedarf der individuellen ärztlichen Beurteilung im Rahmen eines leitliniengerechten Versorgungsprozesses [3, 9]. Dies gilt umso mehr im Hinblick auf den zeitlichen Beginn der Basistherapie. Durch die Einführung des Konzepts der Frühanpassung [5, 11] kann der Beginn der Basistherapie inzwischen deutlich beschleunigt werden. Bereits in den ersten Tagen nach der chirurgischen Implantation kann die Basistherapie in vielen Fällen erfolgreich begonnen werden. Auch wenn dieses Konzept für die Mehrzahl der Patienten anwendbar ist, gilt dies nicht für alle Patienten. Die Frühanpassung ist aber die Voraussetzung für das Erreichen der AHB-Frist von 14 Tagen. Damit ist offensichtlich, dass die überwiegende Anzahl, aber eben nicht alle Patienten geeignet sind, um das AHB-Verfahren als Zugang zur Rehabilitation (Folgetherapie) zu nutzen. Die Feststellung der postoperativen „Rehabilitationseignung“ ist damit eine wichtige, nichtdelegierbare Aufgabe der CIVE, die einen zentralen Beitrag zur Qualitätssicherung der CI-Versorgung liefert. Auch wenn die AHB für die Mehrheit der Patienten als beste Lösung angesehen werden kann, sollten zukünftig auch für die verbleibenden Patienten Strukturen erarbeitet werden, die einen unbürokratischen Zugang zu einer Rehabilitation außerhalb des AHB-Verfahrens ermöglichen.

### Bewertung

Anhand der Ergebnisse der vorliegenden Pilotstudie wurde gezeigt, dass CI-Patienten von einer sehr früh eingeleiteten stationären Rehabilitation (Frankfurter Konzept) profitieren. Durch den vorgezogenen Rehabilitationsbeginn kann eine Wiedereingliederung der Patienten in Beruf und Alltag wesentlich schneller erreicht werden. Des Weiteren reduziert sich durch das vereinfachte AHB-Verfahren der bürokratische Aufwand aller Beteiligten erheblich. Es ergaben sich keine Einschränkungen durch den frühen Beginn der Rehabilitation, insbesondere im Hinblick auf den Effekt der Maßnahme.

### Limitationen der Studie

Das Ziel der vorliegenden Studie war, die Umsetzbarkeit und Effektivität einer sehr frühen stationären CI-Rehabilitation nachzuweisen, um hieraus Rückschlüsse auf die Anwendung des AHB-Verfahrens zu ziehen. Ein motivationsbedingtes Bias ist hierbei nicht auszuschließen, da die Zuordnung zur Studiengruppe durch Patienten selbst vorgenommen wurde, indem diese sich bewusst für oder gegen eine sehr frühe stationäre Rehabilitation in Analogie zu einem AHB-Verfahren entschieden. Darüber hinaus konnten bedingt durch die COVID-19-Pandemie nur wenige Probanden in die Kontrollgruppe eingeschlossen werden. Aus dem gleichen Grund konnten einige Patienten nicht alle regulären klinischen Kontrolltermine wahrnehmen; zusätzlich war bei einigen Patienten aus privaten Gründen die Teilnahme nicht möglich. Die hier vorgestellten Ergebnisse wurden sowohl bei der IG als auch bei der KG im Rahmen einer stationären Rehabilitationsmaßnahme gewonnen. Es kann daher nicht beurteilt werden, ob mit alternativen, z. B. ambulanten Konzepten vergleichbare Ergebnisse erreicht werden können.

## Ausblick

Die Ergebnisse der vorliegenden Pilotstudie legen nahe, die CI-Rehabilitation nach dem Frankfurter Konzept in den Katalog der Anschlussheilbehandlungen aufzunehmen. Gegenwärtig wird eine AHB als eine Leistung zur medizinischen Rehabilitation definiert, die in unmittelbarem zeitlichem Zusammenhang (innerhalb von 14 Tagen) nach einer stationären Krankenhausbehandlung in Anspruch genommen wird. Sie kann im Schnell- bzw. Direkteinleitungsverfahren begonnen werden [8]. Die Aufnahme der CI-Rehabilitation in den AHB-Katalog hätte sowohl für Patienten, Ärzte, Rehabilitationseinrichtungen als auch die Kostenträger enorme Vorteile. Als AHB-Maßnahme würde der Beantragungsprozess deutlich verkürzt und administrativ stark vereinfacht werden. Zudem hätte jeder Patient nach der CI-Op. einen unmittelbaren Anspruch auf eine Rehabilitation, solange diese innerhalb von 2 Wochen nach der Klinikentlassung begonnen werden würde. Durch die Aufnahme der CI-Rehabilitation in den AHB-Katalog würde der leitliniengerechte Versorgungsprozess einheitlich und deutschlandweit umgesetzt werden. Die von einer schweren Hörstörung oder Taubheit betroffenen Patienten würden damit eine wesentlich schnellere Verbesserung ihres Hörvermögens und Sprachverstehens erhalten, was wiederum die berufliche und soziale Wiedereingliederung erheblich beschleunigen könnte. Gerade der letztgenannte Aspekt liegt ebenfalls im unmittelbaren Interesse der Kostenträger. Die mit dem Frankfurter Konzept erhobenen Ergebnisse leisten damit einen wichtigen wissenschaftlichen Beitrag zur Neubewertung der Rehabilitation nach CI-Versorgung als Anschlussheilbehandlung.

## Fazit für die Praxis

- Zum gesamten Prozess der Versorgung mit einem Cochleaimplantat (CI) gehört u. a. die Basis- und Folgetherapie.
- Im Rahmen der Basistherapie erfolgen u. a. medizinische Kontrollen, der Beginn der audiologischen Therapie, die die Erstaktivierung des Prozessors einschließt, sowie hör- und sprachtherapeutische Anteile.
- Es schließt sich die Folgetherapie an, die alle genannten Anteile fortführt, mit Schwerpunkt im Bereich der Hörtherapie und audiologischen Folgetherapie.
- Anteile der Basis- und Folgetherapie können auch in Form einer stationären Rehabilitationsmaßnahme erfolgen.
- Die Rehabilitation ermöglicht ein intensives und individuelles Hörtraining zur Verbesserung der Hörleistung mit dem CI.
- Der Beginn einer stationären Rehabilitationsmaßnahme innerhalb von 2 Wochen nach Implantation eines CI ist erfolgreich umsetzbar.
- Auch bei sehr frühem Rehabilitationsbeginn zeigt sich der positive Effekt einer stationären Rehabilitationsmaßnahme.
- Der Abschluss der Rehabilitation nach etwa 7 Wochen mit guter Verbesserung der Hörfunktion wurde nachgewiesen.
- Die Aufnahme der CI-Rehabilitation in den Katalog der Anschlussheilbehandlungen (AHB) ist wissenschaftlich begründet und damit dringend zu empfehlen.
- Durch die Einführung der CI-AHB würde deutschlandweit für alle Patienten eine standardisierte Versorgungsqualität gewährleistet werden.
